# Association between intrarenal arterial resistance and diastolic dysfunction in type 2 diabetes

**DOI:** 10.1186/1475-2840-7-15

**Published:** 2008-05-23

**Authors:** Richard J MacIsaac, Merlin C Thomas, Sianna Panagiotopoulos, Trudy J Smith, Huming Hao, D Geoffrey Matthews, George Jerums, Louise M Burrell, Piyush M Srivastava

**Affiliations:** 1 Endocrine Centre, Austin Health & University of Melbourne, Melbourne, Australia; 2 Department of Medicine, Austin Health & University of Melbourne, Melbourne, Australia; 3 Diabetes & Metabolism Division, Baker Heart Research Institute, Melbourne, Australia; 4 Vascular Laboratory, Austin Health & University of Melbourne, Melbourne, Australia; 5 Department of Cardiology, Austin Health & University of Melbourne, Melbourne, Australia

## Abstract

**Background:**

In comparison to the well established changes in compliance that occur at the large vessel level in diabetes, much less is known about the changes in compliance of the cardiovascular system at the end-organ level. The aim of this study was therefore to examine whether there was a correlation between resistance of the intrarenal arteries of the kidney and compliance of the left ventricle, as estimated by measurements of diastolic function, in subjects with type 2 diabetes.

**Methods:**

We studied 167 unselected clinic patients with type 2 diabetes with a kidney duplex scan to estimate intrarenal vascular resistance, i.e. the resistance index (RI = peak systolic velocity-minimum diastolic velocity/peak systolic velocity) and a transthoracic echocardiogram (TTE) employing tissue doppler studies to document diastolic and systolic ventricular function.

**Results:**

Renal RI was significantly higher in subjects with diastolic dysfunction (0.72 ± 0.05) when compared with those who had a normal TTE examination (0.66 ± 0.06, p < 0.01). Renal RI values were correlated with markers of diastolic dysfunction including the E/Vp ratio (r = 0.41, p < 0.001), left atrial area (r = 0.36, p < 0.001), the E/A ratio (r = 0.36, p < 0.001) and the E/E' ratio (r = 0.31, p < 0.001). These associations were independent of systolic function, hypertension, the presence and severity of chronic kidney disease, the use of renin-angiotensin inhibitors and other potentially confounding variables.

**Conclusion:**

Increasing vascular resistance of the intrarenal arteries was associated with markers of diastolic dysfunction in subjects with type 2 diabetes. These findings are consistent with the hypothesis that vascular and cardiac stiffening in diabetes are manifestations of common pathophysiological mechanisms.

## Background

Diabetes is associated with the premature development of large vessel arterial stiffness [1]. Increased arterial stiffness is known to be associated with cardiac and kidney dysfunction and is a powerful risk factor for early mortality [2]. In comparison to the well-established changes in compliance that occur at the large vessel level in diabetes, much less is known about the changes in compliance of the cardiovascular system at the end-organ level.

Early in the course of diabetes, progressive cardiac fibrosis and hypertrophy lead impaired relaxation and a stiff left ventricle. We have previously found 'diastolic dysfunction' in over half of all unselected patients with type 2 diabetes attending a diabetes clinic at a tertiary care center [3,4]. Furthermore, recent epidemiological studies show that diastolic dysfunction is an independent risk factor for cardiac death [5]. We have also shown that increased resistance of the intrarenal arteries is associated with a reduced glomerular filtration rate (GFR), an independent predictor of cardiac mortality [6-8].

These findings led us to hypothesize that reduced compliance of the intrarenal arteries that is associated with diabetic kidney disease, is mirrored by changes in the diabetic heart, which are manifested by worsening markers of diastolic function. The aim of this study was therefore to search for a link between compliance of the intrarenal arteries and compliance of the left ventricular wall in individuals with type 2 diabetes.

## Methods

### Subjects

We studied 167 unselected patients with type 2 diabetes attending the diabetes clinics at the Endocrine Centre, Austin Health, Australia. The Austin Health Human Research Ethics Committee approved the research described in this study and informed consent was obtained from all participants.

### Kidney duplex scans

Intra-renal vascular resistance was estimated by renal duplex ultrasound as previously described [6]. The renal resistance index (RI) was calculated as the peak systolic velocity – end diastolic velocity)/peak systolic velocity. The upper end of the normal range for intra-renal RI values in healthy subjects is < 0.7 [9]. Individuals with an RI > 0.7 were said to have increased intra-renal vascular resistance

### Transthoracic echocardiograms

Transthoracic echocardiogram (TTE) was performed using a commercially available ultrasound system (Acuson Sequoia, 3.5 Mhz transducer) in the left lateral decubitus position and methods that have been described previously [3,4]. Recordings and measurements were obtained according to the published recommendations of the American Society of Echocardiography [10,11] and analysed by two experienced, independent observers. Conventional parameters of diastolic function were measured at the mitral leaflet tips over three consecutive cardiac cycles. In the apical four chamber view, a 2 mm pulse wave Doppler sample gate was placed at the medial mitral annulus to obtain the peak early diastolic (E'), and used to calculate the E/E' ratio. Diastolic function was classified according to the Canadian consensus on diastolic dysfunction [12]. Systolic dysfunction was defined by evidence of regional wall motion abnormalities and/or an ejection fraction of less than 50%.

### Clinical and biochemical measurements

An isotopic glomerular filtration rate (iGFR) was measured by the plasma disappearance of isotopic ^99m^Tc-diethylene-triamine-penta-acetic acid (DTPA) employing the Brochner-Mortensen correction. Glycated haemoglobin (HbA_1c_), fasting lipids, serum electrolytes and urinary albumin excretion (AER) were measured as previously described [6,13]. A full clinical and medication history including the presence or absence of diabetic and cardiovascular complications, and treatment history was also recorded for each participant.

### Statistics

Continuous data are expressed as mean ± standard deviation (SD). Categorical data are expressed as frequency (%). Where appropriate, means and proportions were compared by using analysis of variance and chi-square tests, respectively. The Pearson method was used to test for a correlation between the intrarenal RI and the echocardiographic parameters of diastolic function. A multivariate linear regression model was used to confirm the contribution of independent association between RI and cardiac parameters. All variables known to be associated with diastolic function were included in the final model, along with any variables associated with diastolic function in univariate analyses with a p value < 0.01. The potential for multiple colinearity was tested using the variance inflation factor (VIF) and condition number (CN), where VIF <10 and CN <30 are desirable.

## Results

The clinical and biochemical characteristics of the study population are shown in Table 1. Many clinic subjects were obese (57%), had hypertension (81%) or established cardiovascular disease (36%). Over half of all patients in this cohort had chronic kidney disease (CKD) including elevated AER levels (>20 μg/min) in 45% and 22% with an iGFR < 60 ml/mim/1.73 m^2^.

**Table 1:** Clinical and biochemical parameters for 167 subjects with type 2 diabetes, stratified according to cardiac function

	Total cohort (n = 167)	Normal cardiac function (n = 59)	Diastolic dysfunction alone (n = 66)	Diastolic and systolic dysfunction (n = 30)
Intrarenal RI	0.69 ± 0.01	0.66 ± 0.06	0.72 ± 0.06**	0.71 ± 0.06**
Age (years)	62 ± 12	55 ± 13	66 ± 9*	66 ± 12*
Sex (% male)	59%	59%	54%	72%
Diabetes duration (years)	11 ± 8	9 ± 6	13 ± 8*	10 ± 7
Obese BMI (>30 kg/m^2^, %)	57%	48%	60%	56%
History of CVD (%)	36%	23%	32%	69%*
HbA_1c _(%)	7.7 ± 1.5	8.1 ± 1.5	7.6 ± 1.3*	7.4 ± 1.9*
Metformin use(%)	68%	80%	55%*	69%
Sulphonylurea use (%)	57%	62%	50%	72%
Glitazone use (%)	19%	31%	11%*	17%
Insulin use (%)	42%	45%	46%	24%
Hypertension (%)	81%	69%	85%*	90%*
Systolic BP (mmHg)	140 ± 17	135 ± 17	144 ± 18	141 ± 15
Diastolic BP (mmHg)	75 ± 8	76 ± 8	74 ± 8	76 ± 9
RAS inhibitor use (%)	72%	54%	84%*	83%*
Number of antihypertensives (n)	1.9 ± 1.4	1.3 ± 1.3	2.1 ± 1.4*	2.7 ± 1.5*
Total cholesterol (mM)	4.7 ± 0.9	4.9 ± 1.0	4.8 ± 0.8	4.6 ± 1.0
Triglycerides (mM)	2.1 ± 1.2	2.3 ± 1.3	2.0 ± 1.1	2.3 ± 1.4
Treatment for dyslipidaemia	63%	58%	53%	90%*
History of retinopathy	27%	14%	44%*	17%
iGFR (ml/min/1.73 m^2^)	93 ± 32	111 ± 27	82 ± 27*	73 ± 30*
GFR< 60 (ml/min/1.73 m^2^)	22%	6%	25%*	39%*
AER > 20 (μg/min)	45%	41%	39%	59%*

Data for continuous variables are mean ± SD. Data for categorical variables are expressed as proportions.

* p < 0.05, ** p < 0.01 vs individuals with normal cardiac function on TTE,

Subjects were further stratified according to results of their TTE examination. Thirty five percent of participants had normal cardiac function on TTE examination (n = 59). However, the majority of diabetic subjects (65%) had abnormal cardiac function (n = 108), including 66 individuals with diastolic dysfunction alone (40%), 30 with concurrent diastolic and systolic dysfunction (18%) and in a small number of individuals (n = 12) isolated systolic dysfunction was the only finding (7%). Subjects with diastolic dysfunction were generally older, had a greater frequency of hypertension and renal impairment than those with a normal TTE examination. As expected, subjects with concurrent systolic dysfunction had a greater prevalence of past CVD (Table 1).

**Figure 1:**
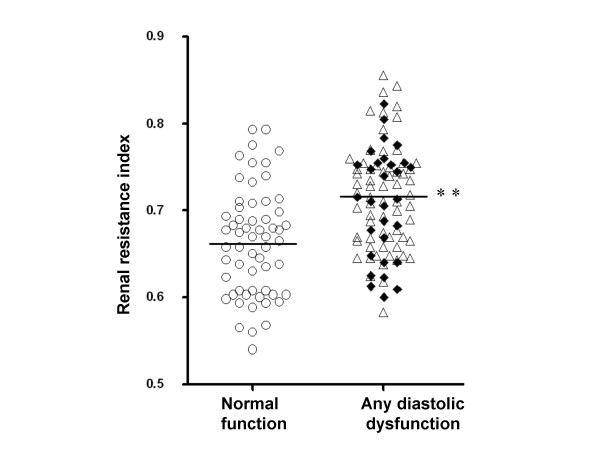
**Intrarenal resistance index values in subjects with type 2 diabetes, stratified according to cardiac function on a transthoracic echocardiogram. **○ denotes individuals with normal cardiac function. △ denotes individuals with diastolic dysfunction alone. ♦ denotes individuals with diastolic and systolic dysfunction. ** P < 0.01 vs normal transthoracic echocardiogram.

The mean renal RI was 0.69 ± 0.01 in the total cohort with 48% of subjects with type 2 diabetes having an elevated RI beyond the normal range (> 0.7). As shown in Figure 1, renal RI values were significantly higher in with (any) diastolic dysfunction (0.72 ± 0.06) compared with those who had a normal TTE examination (0.66 ± 0.06, p < 0.01). Moreover, the finding of an elevated RI level (> 0.7) was twice as common in individuals with (any) diastolic dysfunction compared with those who had a normal TTE examination (59% vs 25%, p < 0.001). Less than 18% of all those with elevated RI levels (> 0.7) in this cohort had normal cardiac function. As shown in Table 1, RI levels were elevated to a similar extent in subjects with diastolic dysfunction alone (0.72 ± 0.06) and in those with concurrent diastolic and systolic dysfunction (0.71 ± 0.06) when compared those who had a normal TTE examination (0.66 ± 0.06, both p < 0.01).

**Figure 2:**
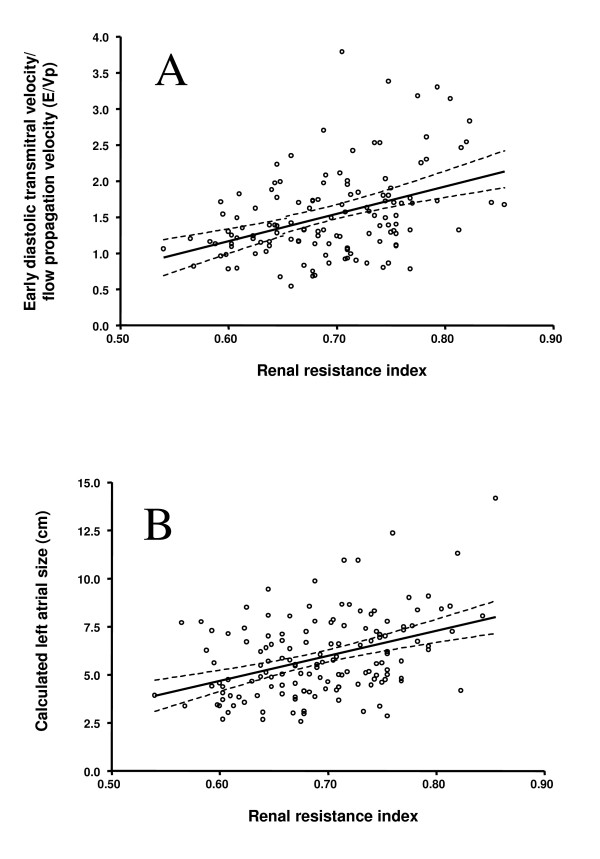
Correlations between the intrarenal resistance index and markers of diastolic dysfunction, i.e., (A) the E/Vp ratio (r = 0.41, p < 0.001) and (B) left atrial area (r = 0.36, p < 0.001). Solid lines denote the linear regression relationship between the variables. The dotted lines denote the 95% confidence interval for the regression relationship.

As shown in figure 2, renal RI values were correlated with markers of diastolic dysfunction including the E/Vp ratio (r = 0.41, p < 0.001) and left atrial area (r = 0.36, p < 0.001). Renal RI values were also associated with the E/A ratio (r = 0.36, p < 0.001) and the E/E' ratio (r = 0.31, p < 0.001). The association between renal RI levels and all of the above markers of diastolic dysfunction remained significant (all p <0.001) after correcting for multiple clinical and biochemical variables including age, duration of diabetes, BMI, blood pressure, albumin excretion rate, iGFR levels, use of blockers of the renin-angiotensin system, as well as adjusting for parameters of systolic function. While renal RI values were weakly correlated with parameters of systolic function (e.g fractional shortening and ejection fraction, both p = 0.05), these associations were eliminated after adjusting for age.

## Discussion

This study shows that resistance of the intrarenal arteries is closely linked with abnormal cardiac function in patients with type 2 diabetes. In particular, there was a continuous relationship between increasing resistance of the intrarenal arteries and established indices of left ventricular filling pressure and diastolic function. These associations were independent of systolic function, hypertension, the presence and severity of chronic kidney disease, the use of renin-angiotensin inhibitors and other potentially confounding variables.

These data support the hypothesis of a common pathway to diabetic complications, which promotes stiffening and progressive dysfunction in both the heart and major vessels. Such common contributory factors may include oxidative stress, insulin resistance, inflammation, the accumulation of advanced glycation end products (AGEs), activation of the rennin angiotensin system (RAS), arterial calcification and endothelial (dys)function. However, it is likely that a parallel increase in the resistance of the intrarenal arteries and indices of diastolic dysfunction also partly reflects a generalized reduction in vascular compliance associated with arteriosclerosis in a range of vascular beds. In support of this suggestion, both the intra-renal RI and parameters of diastolic dysfunction have been shown to be associated with systemic pulse wave velocity, arterial compliance, and systemic vascular damage in both diabetic and non-diabetic subjects [14,15].

### Limitations

One of the weaknesses of this study was that we did not measure total arterial compliance and that we have used the intrarenal RI and indices of diastolic dysfunction as surrogate markers of intrarenal arterial and ventricular compliance, respectively. It is appreciated that functional as well as structural parameters may have influenced our results. Also, we did not account for the possibility of the presence of an elevated central venous pressure or autonomic neuropathy, factors that may have contributed to the development of both intrarenal arterial compliance and diastolic dysfunction.

## Conclusion

This study demonstrates that worsening indices of diastolic function in subjects with type 2 diabetes parallels increases in resistance of the intrarenal vessels. These findings are consistent with the hypothesis that both the development of vascular and myocardial stiffening in diabetes are manifestations of common pathophysiological mechanisms. It is possible that these processes also contribute to the link between renal dysfunction and cardiovascular outcomes in subjects with type 2 diabetes [7,8].

## Abbreviations

TTE: transthoracic echocardiogram; RI: resistance index; iGFR: isotopic glomerular filtration rate (measured by the plasma disappearance of ^99m^Tc-DTPA employing the Brochner-Mortensen correction); E: early mitral flow velocity; E': early diastolic velocity of the mitral annulus; A: late mitral flow velocity; Vp: flow propagation velocity; BMI: body mass index; BP: blood pressure; RAS: renin angiotensin system; CHD: coronary heart disease; AER: albumin excretion rate; CRP: C-reactive protein; HbA_1c_: glycated haemoglobin.

## Competing interests

The authors declare that they have no competing interests.

## Authors' contributions

RJM drafted the paper. RJM, SP, TJS, PGM, MCT, GJ, LMB & PMS were involved in the study design, coordination and data acquisition. MCT performed the statistical analysis of the data presented. LMB & PMS coordinated and interpreted the TTE studies whilst PGM & HH coordinated and interpreted the renal arterial RI studies. All authors approved the final version of the manuscript.
